# *Lin-28* Regulates Oogenesis and Muscle Formation in *Drosophila melanogaster*

**DOI:** 10.1371/journal.pone.0101141

**Published:** 2014-06-25

**Authors:** Vassilis Stratoulias, Tapio I. Heino, Frederic Michon

**Affiliations:** 1 Department of Biosciences, University of Helsinki, Helsinki, Finland; 2 Institute of Biotechnology, Developmental Biology Program, University of Helsinki, Helsinki, Finland; Technische Universität Dresden, Germany

## Abstract

Understanding the control of stem cell (SC) differentiation is important to comprehend developmental processes as well as to develop clinical applications. Lin28 is a conserved molecule that is involved in SC maintenance and differentiation by regulating *let-7* miRNA maturation. However, little is known about the *in vivo* function of Lin28. Here, we report critical roles for *lin*-28 during oogenesis. We found that *let*-7 maturation was increased in *lin-28* null mutant fly ovaries. We showed that *lin-28* null mutant female flies displayed reduced fecundity, due to defects in egg chamber formation. More specifically, we demonstrated that in mutant ovaries, the egg chambers fuse during early oogenesis resulting in abnormal late egg chambers. We also showed that this phenotype is the combined result of impaired germline SC differentiation and follicle SC differentiation. We suggest a model in which these multiple oogenesis defects result from a misregulation of the ecdysone signaling network, through the fine-tuning of *Abrupt* and *Fasciclin2* expression. Our results give a better understanding of the evolutionarily conserved role of *lin-28* on GSC maintenance and differentiation.

## Introduction

The Cold-Shock Domain (CSD) protein Lin28 was initially identified in *Caenorhabditis elegans* (*C. elegans*) as a component of the heterochronic pathway that regulates the timing of cell fate specification [1]. Subsequent discovery of gene expression regulation through small non-coding RNAs clarified the role of Lin28 in this pathway [2]. The *lin-28* mRNA is a conserved target of the *let-7* micro-RNA (miRNA) family both in *C. elegans* and vertebrates [3], [4]. On the other hand, Lin28 inhibits *let-7* processing [5]. At the molecular level, Lin28 protein interacts with the *let*-7 precursor (pre-let-7), resulting in inhibition of *let*-7 maturation [6]. The *let-7* inhibition occurs through the physical interaction of the pre-*let*-7 loop and Lin28 protein, preventing further processing of *pre-let-7* towards the mature form of *let-7* [7], [8]. Together, these interactions create a feedback loop between Lin28 and *let-7*, leading to a strict regulation of *let-7* maturation [9].

Lin28 raised further interest when it was used, along with Nanog, to replace the factors c-Myc and Klf4 in somatic cell reprogramming [10]. These experiments, together with data from human embryonic stem cells [11], underscored the important role of *lin-28* in pluripotency regulation and maintenance. Besides acting as a negative regulator of *let-7* maturation, Lin28 has also been shown to have a direct effect on translation through the recruitment of the RNA Helicase A [12]. This mode of function, independent of *let-7* maturation, has been demonstrated in the case of Insulin-like Growth Factor 2 during mouse myogenesis. Lin28 binding on IGF-2 mRNA increases its translation efficiency and therefore facilitates skeletal myogenesis in mice [13].

The Lin28 protein is composed of four domains: a positively charged linker that binds two Cys-Cys-His-Cys (CCHC)-type zinc-binding motifs to the CSD. In mammalian genomes, two paralogs of *lin-28* are found, *Lin28A* and *Lin28B.* While Lin28B represses *let-7* processing in the nucleus to prevent the formation of the precursor form from the primary *let-7*, Lin28A also blocks cytoplasmic processing of let-7 [6]. It has recently been shown in mouse that deletion of the Lin28 linker domain alters the protein’s three-dimensional structure and is sufficient to disrupt sequestration of the precursor form of *let-7* (*pre-let-7*) [14].

The miRNA *let-7* family is conserved across diverse animals, functioning to control late temporal transitions during development [15]. During the last decade, the involvement of *let-7* in regulating cell differentiation has been analyzed in various contexts, including neural cell specification, stem cell maintenance and hematopoietic progenitor differentiation [16]–[18]. While eight different *let-7* miRNA genes are annotated in the human genome, only one is found in *Drosophila melanogaster* (for review, [19]). Like in *C. elegans*, in *Drosophila* the loss of *let-7* expression leads to the modification of temporal regulation of the metamorphosis process [20]. During fly metamorphosis, the expression of *let-7* complex (*let-7C*), a polycistronic locus encoding the *let-7*, *miR-100* and *miR-125* miRNAs, is under direct control by the steroid hormone ecdysone. Ecdysone is the central regulator of insect developmental transitions [21]. Therefore, *let-7* has been proposed to be part of a conserved, ecdysone regulated pathway that controls the timing of the larva to adult transition [22].

In addition to affecting the metamorphosis clock, Sokol and colleagues have shown that the *let-7* deletion also affects the neuromuscular remodeling that takes place during the larva to adult transition [23]. During neuromuscular remodeling, and under normal conditions, the dorsal internal oblique muscles (DIOMs) disappear 12 hours after emergence of the adult fly from the pupa. However, the adult *let-7* mutants retain the DIOMs through adulthood. Deletion of the *let-7* gene is sufficient to induce this phenotype, while deletion of either *miR-100* or *miR-125* genes is not enough to recapitulate the DIOM phenotype. Furthermore, *let-7* has been shown to govern the maturation of neuromuscular junction of adult abdominal muscles, through regulation of *Abrupt* expression [20].

While previous studies have demonstrated that the *let-7* target Abrupt and ecdysone signaling are required for oogenesis in fruit fly ovaries [24], and that the *let-7* miRNA family is abundantly expressed both in newborn mouse ovaries [25] and in fly ovaries [21], no study has been conducted on the role of Lin-28/*let-7* network in *Drosophila* ovaries. Therefore, we decided to study the effects of *lin-28* during *Drosophila melanogaster* development from the egg to the adult, and more particularly during oogenesis.

We generated a *lin-28* mutant and validated the consequent increase of *let-7* maturation. We found that *lin-28* knockout resulted in reduced muscular performance and defects in DIOM morphogenesis. These results were in line with the *let-7* knock out muscular phenotype described earlier [23]. Moreover, we identified multiple defects during oogenesis due to abnormal follicle and germline stem cell (FSCs and GSCs respectively) differentiation. We propose a link between ovarian defects and ectopic expression of *Fascicilin2* (*Fas2*), a known downstream target of the Ecdysone pathway [26], and a predicted *let-7* target.

## Materials and Methods

### Fly Stocks and Genetics

Flies were raised on standard food at 25°C on a 12 hour light 12 hour dark cycle. The following stocks were used: *P{EP}lin28^EP915^* (Bloomington Drosophila Stock Centre (BDSC)#17298), *P{Δ2–3}* (BDSC#3629), *Df(3L)Exel6106* (BDSC#7585), *Df(3L)ZN47* (BDSC#3096), *da-Gal4* [27], *UAS-nGFP* (BDSC#4775), *UAS-let-7* (BDSC#41171), *Sb*/TM3 *ActGFP Ser* (BDSC#4534). *w^1118^* flies were regarded as wild type.

### Generation of *lin-28* Mutant Alleles

One hundred *lin28* alleles were generated by imprecise excision from *P{EP}lin28^EP915^*. Two lines, namely *lin28^dF30^* and *lin28^dF101^*, were selected and further characterized by PCR and sequence analysis.

### Quantitative Real-Time PCR

Total RNAs were isolated from 20 ovaries of each genotype as previously described [28]. RNAs were reverse-transcribed into cDNAs using the GenoExplorer miRNA First-Strand cDNA Core Kit (GenoSensor Corporation). The expression of *pre-let-7* and *let-7* were analyzed by using the GenoExplorer miRNA First-Strand cDNA Core Kit (GenoSensor Corporation). The *pre-let-7* and mature *let-7* specific primers were purchased from GenoSensor Corporation. U6 snRNA was used as reference gene in order to normalize microRNA expression. All qRT-PCR were carried out independently five times.

The ratio of gene or microRNA expression was compared to the internal control and was calculated based on the formula 2∧ (Cpcontrol – Cpsample).

### Spontaneous Locomotion Activity Assay

Single, 4-day-old male flies were anesthetized with CO_2_, transferred to 1 cm^3^ chambers and allowed to recover for 30 min. Locomotion was quantified as the number of times the fly walked across the midline of the chamber over a 2 minute period [23], [29].

### Induced Locomotion Activity Assay

The locomotor ability was determined with a negative geotaxis assay as described previously [30]. Ten 4-day-old male flies were anesthetized with CO_2_ and put in a volumetric glass cylinder (opening width = ∼2.7 cm; height of cylinder shaft = 24 cm). The cylinder was graded with masking tape at 2 cm and 20 cm from the base of the cylinder, resulting in three areas (bottom, middle and top respectively). After transfer to the cylinder, flies were allowed 30 minutes to recover. The cylinder was tapped gently causing the flies to fall to the bottom and after 1 minute the number of flies within the three different areas was scored. Each experiment was repeated 3 times.

### Oviposition Assay

Newly eclosed females (emerging from pupae) were collected and mated with wild type males in new vials containing laboratory standard food supplemented with dry yeast. At day seven after emerging from pupae, the flies were transferred to new food vials with supplemented dry yeast. At day 10 after emerging from pupae single females were put on apple juice plates with added dry yeast for 22 hours. Subsequently, the number of eggs on the apple juice plates was counted. For heterozygous flies, the number of GFP positive eggs was scored under a GFP microscope (Leica MZ-FLIII).

### Immunohistochemistry

Adult abdomens were prepared as previously described [23]. Ovaries were dissected in phosphate-buffered saline (PBS) and fixed while shaking on a nutator for 20 min in PBS containing 4% formaldehyde (Fluka#47630). Next, they were rinsed two times and subsequently washed three times for 20 min in PBT (PBS/0.1% Triton X-100). Tissues were blocked for 30 minutes in 5% Normal Goat Serum (NGS; Jackson ImmunoResearch) in PBT and incubated with primary antibodies overnight at 4°C. Ovaries were rinsed twice, washed three times for 20 min with PBT and incubated in secondary antibodies in 5% NGS in PBT overnight at 4°C. Next, tissues were rinsed three times in PBT, followed by two rinses for 20 min in PBS and finally were stored in PBS until microscopy. The following antibodies from Developmental Studies Hybridoma Bank were used: mouse anti-EcR (1∶10, DDA2.7), mouse anti-Fas2 (1∶500, 1D4), mouse anti-hts-RC (1∶30), mouse anti-orb (1∶20, 4H8) and mouse anti-spectrin (1∶30). In addition, we used rabbit anti-phosphohistone-3 (1∶1000) (Upstate Cells Signaling Solutions #06-570). Secondary antibodies used were AlexaFluor 488 conjugated goat anti-rabbit IgG (H+L) (1∶1000, Molecular Probes), DyLight 549- and DyLight 649-conjugated F(ab′)_2_ fragments goat anti-mouse IgG (H+L) and DyLight 633-conjugated F(ab′)_2_ fragments goat anti-rat IgG (1∶200, Jackson Immunoresearch). Additional stains include rhodamine phalloidin (1∶1000, Sigma). Samples were mounted in Vectashield with or without DAPI (Vector Laboratories). Images were obtained with a confocal laser-scanning microscope (Leica TCS SP5) and processed with Image J and Adobe Photoshop. All confocal images presented are sections, apart from Figure 3, Figure 5A to 5B’ and Figure 6C to 6D” that are z-stacks.

## Results

### Lin-28 Blocks let-7 Maturation in *Drosophila* Ovaries

Imprecise P-element excision was used to generate deletions of the *lin-28* gene from the homozygous viable P-element line *lin^[EP915]^*. Two hundred mutant flies with potential excision were created, all of which were viable and fertile as homozygous. Homozygous mutant animals exhibited low fecundity and abnormal motility compared to heterozygous animals. We selected two independent excision lines, hereafter referred as *lin-28^dF30^* and *lin-28^dF101^*, which we further characterized.

**Figure 1:**
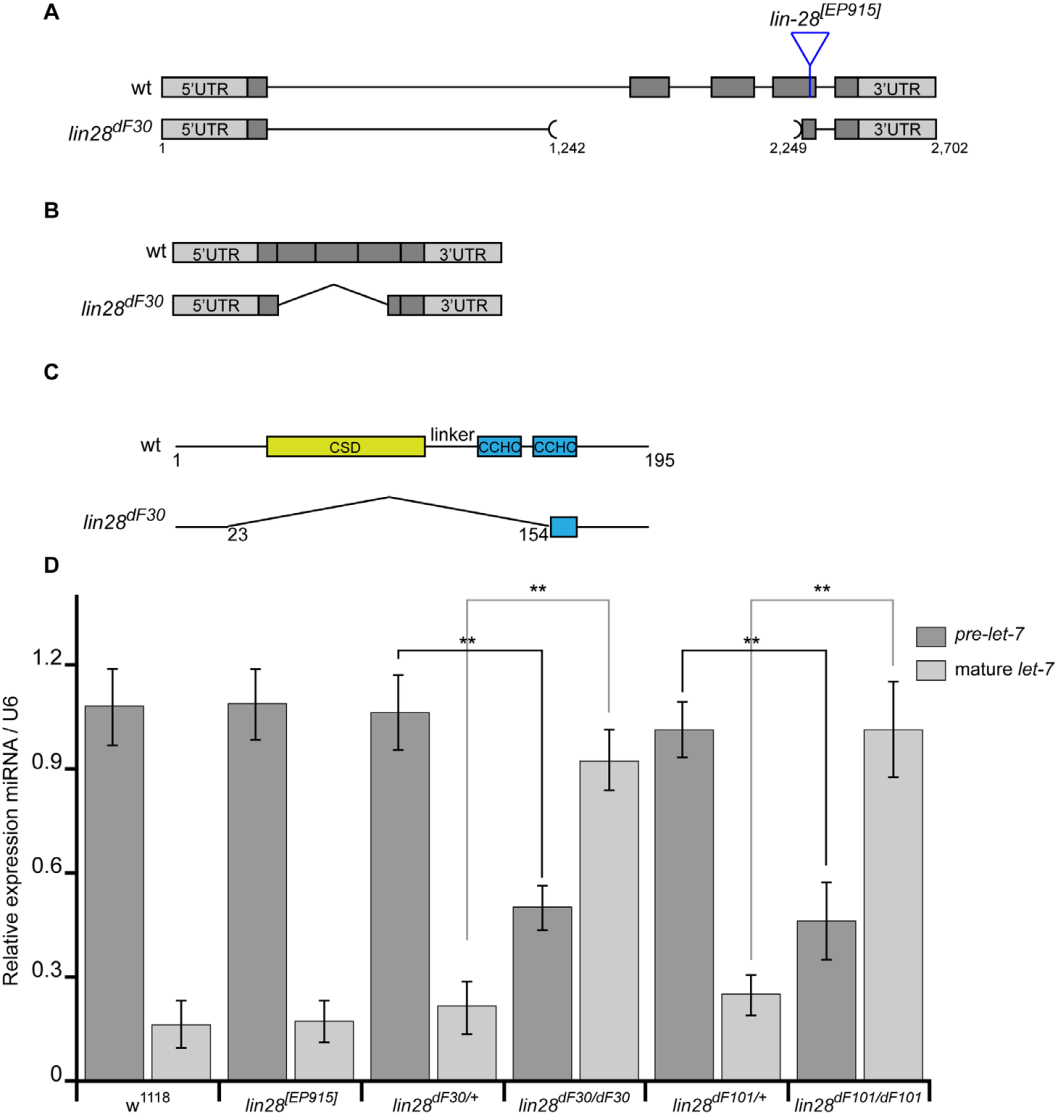
Characterization of the *lin-28^dF30^* Allele. (**A**) The *lin-28^dF30^* allele was created by imprecise P-element excision that resulted in a deletion of 1007 bases, upstream of the P-element insertion. (**B**) The predicted *lin-28^dF30^* mRNA lacked the second, the third and partially the fourth exon. (**C**) The predicted Lin-28^dF30^ protein lacked the CSD, the linker and at least one of the CCHCs. (**D**) qRT-PCR analysis of *Drosophila* ovaries, demonstrated that the control of *pre-let-7* processing is greatly affected by the deletion of *lin-28* compared to controls, resulting in a dramatic increase in the levels of mature *let-7* (from 0.17 in *w^1118^*, 0.18 in *lin-28^[EP915]^*, 0.21 in *lin-28^dF30/+^* and 0.23 in *lin-28^dF101/+^* to 0.92 in *lin-28^dF30^* and 0.95 in *lin-28^dF101^* mutant flies). Student t-test: p<0.05.

The P-element excision in the *lin-28^dF30^* allele resulted in deletion of 1,007 bp upstream of the original P-element position (Figure 1A). The resulting *lin-28^dF30^* mRNA lacked the second and third exons, as well as part of the fourth exon (Figure 1B). Therefore, the predicted Lin28^dF30^ protein would lack the CSD, the linker and the first CCHC domain (Figure 1C). Recently, it has been shown that physical interaction between Lin28 and *let-7* requires both the 3′ end of the CSD, as well as the linker domain [14]. Thus, we consider *lin-28^dF30^* as a null allele. We also analyzed a second allele, *lin-28^dF101^*, which was chosen due to phenotypic similarities with *lin-28^dF30^*. The imprecise P-element excision led to a deletion of 13 bp upstream the P-element insertion site. The resulting predicted Lin28^dF101^ protein was 32 aminoacids shorter due to a premature stop codon in the second CCHC domain. Moreover, this domain would present 10 aminoacid residues different from the original sequence. While we could not consider *lin-28^dF101^* as a null allele, we used it as second mutated allele. In both mutant chromosomes the *lin-28* neighbouring genes, such as *Sse* and *Blimp1*, were not affected.

To determine whether the *lin-28* deletion affects *let-7* maturation, we investigated the *let-7* expression and maturation in *Drosophila* ovaries, a tissue where *let-7* expression has been reported before [21]. As Lin28 regulates *let-7* maturation, we performed quantitative PCR to evaluate the *pre-let-7* and the mature *let-7* content in fly ovaries. We used two *lin-28* generated alleles to rule out a secondary genomic effect due to the imprecise deletion, *lin-28^dF30^* and *lin-28^dF101^*. We compared the data acquired from the homozygous mutant ovaries, with data from wild type (*w^1118^*), homozygous *lin^[EP915]^* (P element line used for original excisions) and heterozygous (*lin-28^dF30/+^* and *lin-28^dF101/+^*) ovaries and we found that in *Drosophila* ovaries, *let-7* maturation is tightly regulated by Lin28 and that the loss of Lin28 activity disturbed this regulation. In more details, we found that in ovaries from wild type line (*w^1118^*), homozygous *lin^[EP915]^* and heterozygous (*lin-28^dF30/+^* and *lin-28^dF101/+^*) animals, only a small part of *pre-let-7* gave rise to mature *let-7*. In addition, these results demonstrated that in the *lin^[EP915]^* line (the P element line used for original excisions) the Lin28 gene function is not impaired and the *let-7* processing occurs as in the other controls (Figure 1D). In contrast, deletion of *lin-28* (*lin-28^dF30/dF30^* and *lin-28^dF101/dF101^*) resulted in a dramatic increase of *let-7* maturation. Notably, we detected a shift in the proportion of mature *let-7* versus *pre-let-7* in homozygous *lin-28^dF30^* ovaries, reflecting the importance of Lin28 inhibitory action on *pre-let-7* maturation. Consistent with the *let-7* quantification, we detected *let-7* ectopic maturation in homozygous *lin-28^dF30^* ovaries by *in situ* hybridization (data not shown).

### *lin-28* Deletion Leads to Muscle Defects in Adult Flies

Previous studies have demonstrated the involvement of *let-7* in the formation of neuromuscular junctions and proper adult muscle remodeling [20], [23]. Therefore, the published data constitute an interesting comparison to our newly created *lin-28* mutant line.

**Figure 2:**
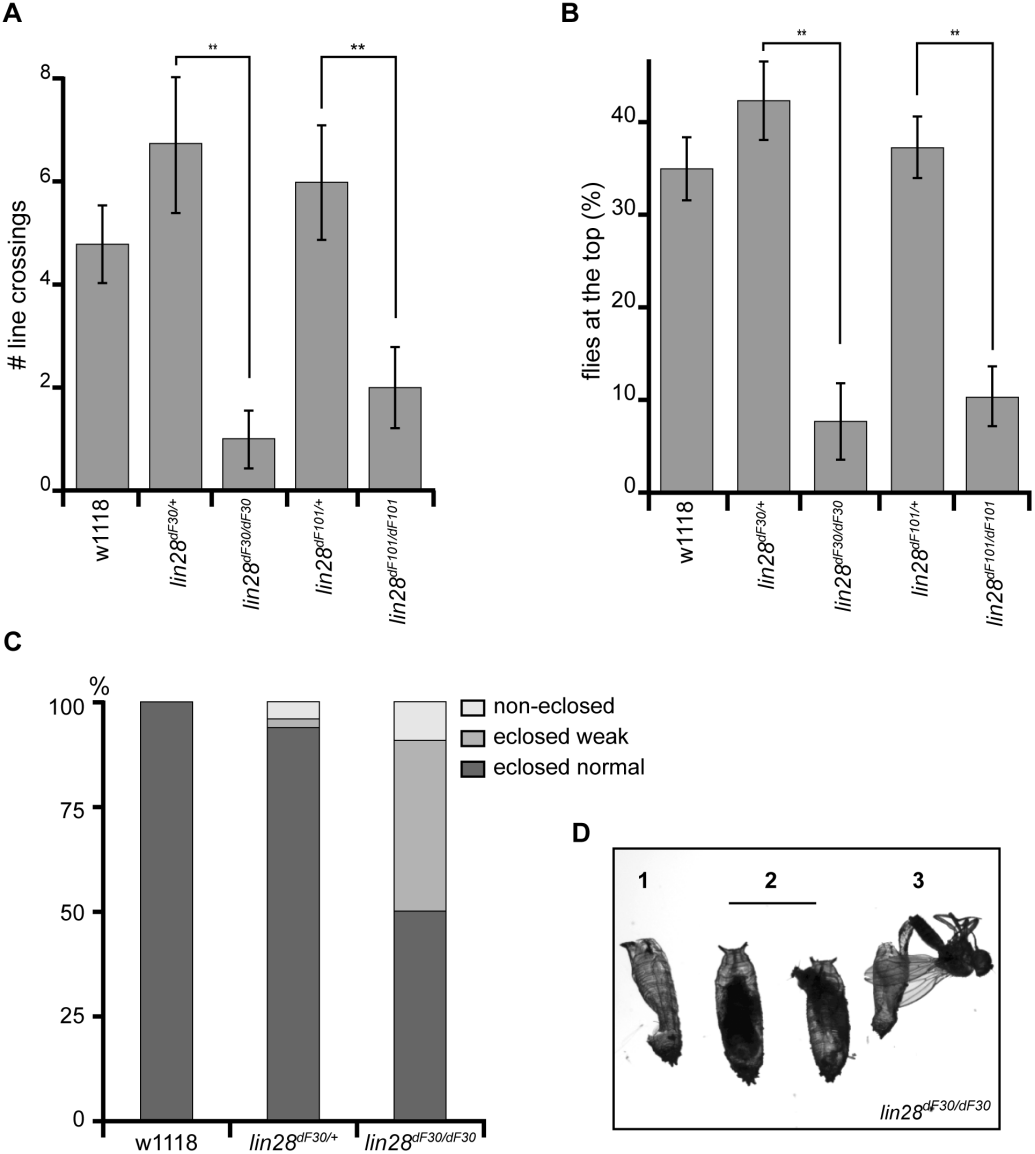
Lin28 is Required for Proper Geotaxis and Eclosion. *lin-28^dF30^* and *lin-28^dF101^* mutants exhibited reduced levels of both spontaneous (**A**) and stimulated (**B**) locomotory activity (n = 50; p<0.05). (**C**) 50% of the homozygous *lin-28^dF30^* mutants either did not eclose (41%), or eclosed too weak to survive (9%), while 100% of the *w^1118^* and 94% of the *lin-28^dF30/+^* progeny eclosed properly (*w^1118^* n = 43; *lin-28^dF30/+^* n = 44; *lin-28^dF30^* n = 51). (**D**) Example of an empty pupal case (**1**), reflecting a full eclosion, while some mutant flies did not complete their eclosion (**2**) or were too exhausted to survive the eclosion process (**3**)

We monitored the spontaneous (Figure 2A) and induced (Figure 2B) locomotion of 4 day-old male flies from five different genotypes: homozygous *lin-28^dF30^* and *lin-28^dF101^*, heterozygous *lin-28^dF30/+^* and *lin-28^dF101/+^*, and *w^1118^* (n = 50 for each phenotype). Both tests displayed a drastic loss of locomotion when comparing heterozygous and homozygous flies. While the heterozygous flies behaved like the wild type control line (*w^1118^*), the mutant *lin-28^dF30^* and *lin-28^dF101^* individuals were less prone to walk spontaneously (Figure 2A), or upon stimulation (Figure 2B). We noticed similar results with flight assays (data not shown).

**Figure 3:**
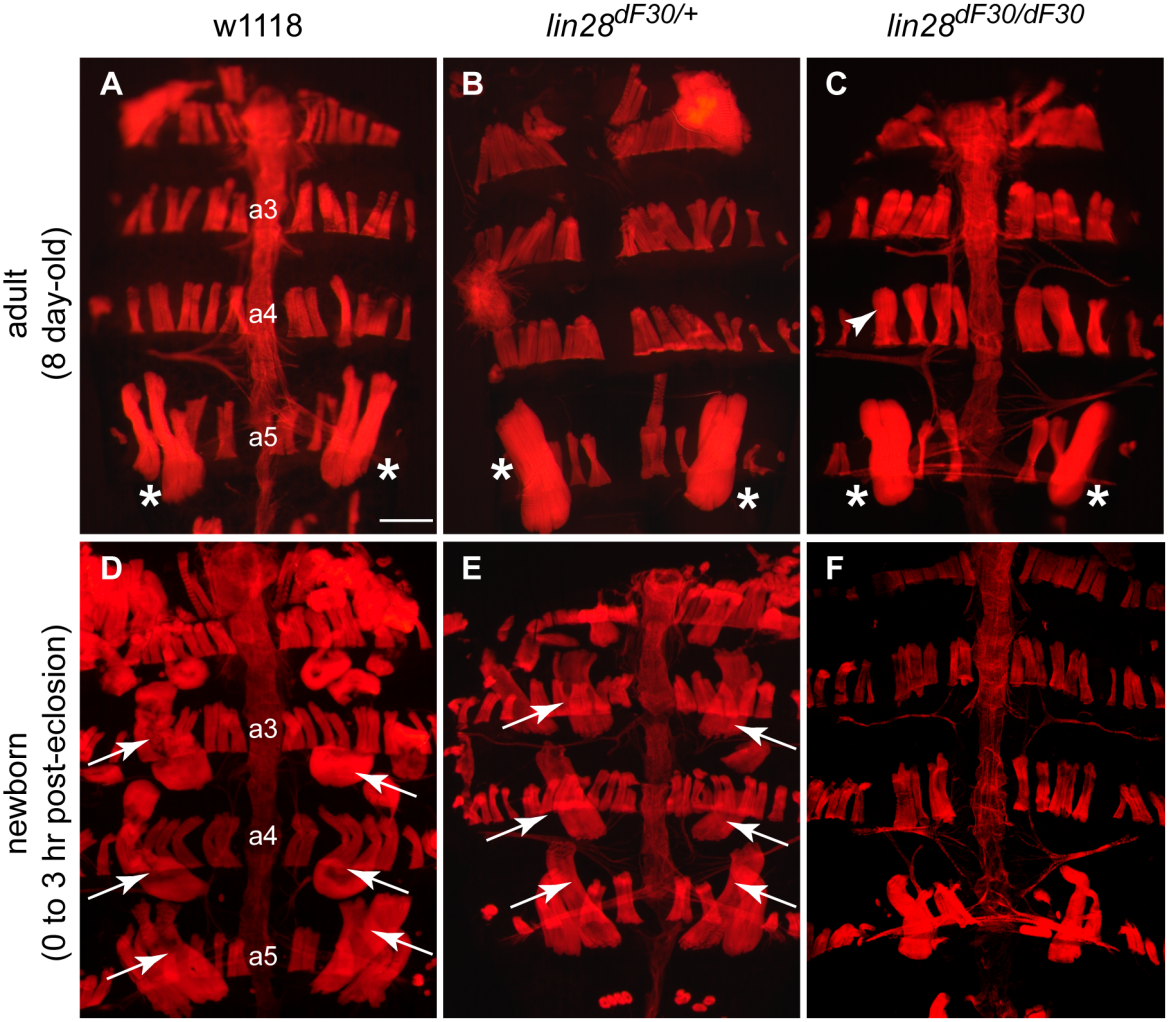
*lin-28^dF30^* Newborn Flies Lack DIOMs. (**A**, **B**) 8-day old *w^1118^* (n = 7) and *lin-28^dF30/+^* males (n = 10) have a bilaterally symmetrical male specific muscle, which spans the segment a5 of the abdomen, called muscle of Lawrence (asterisks). (**C**) 8-day old male *lin-28^dF30^* mutant (n = 10), did not present any dramatic muscle phenotype. However, they exhibited minor defects such as shorter muscle of Lawrence (asterisk) and larger dorsal muscles (arrowhead). (**D**, **E**) Newly eclosed *w^1118^* (n = 3) and *lin-28^dF30/+^* males (n = 5) have the transitory muscles called DIOMs (arrows). (**F**) Newly eclosed *lin-28^dF30^* mutants (n = 5) presented a loss of the DIOMs. Scale bar represents 100 µm.

Our results clearly pointed towards a muscle defect due to *lin-28* deletion. However, these behavioral tests were biased. We noticed that up to 50% (n = 51) of homozygous *lin-28^dF30^* flies either failed to eclose or died shortly after their eclosion (Figure 2C and 2D, Movie S1). These failures represented only 7% (n = 44) of the heterozygous *lin-28^dF30/+^* fly progeny. Therefore, performing geotaxis experiments on 4 day-old males disregarded the the most severe *lin-28^dF30^* phenotype. Consequently, we decided to compare the abdominal muscle morphology of adult flies and late pupae (Figure 3). It has been previously shown that loss of *let-7* results in a drastic DIOM phenotype. DIOMs are the muscles which are required to exit the pupal case and which are lost within 12 hours after eclosion [23]. In particular, *let-7* mutants retained DIOMs at various frequencies, even two days after eclosion, while they also exhibit minor morphological defects in dorsal muscles. Consistently, 8 day-old male homozygous *lin-28^dF30^* flies did not exhibit obvious muscle defects. However, we noticed slight changes in abdominal muscle morphology of *lin-28^dF30^* flies (Figure 3A–C - arrowhead). These changes may explain the decrease of strength in the spontaneous and upon stimulation locomotion assays.

Because of the importance of DIOMs during eclosion, we monitored their structure upon *lin-28* mutation. The newly eclosed heterozygous *lin-28^dF30/+^* animals exhibited a pair of DIOMs on the abdominal segments a2, a3, a4 and a5 (Figure 3D and 3E - arrows; n = 5). Strikingly, the newly eclosed homozygous *lin-28^dF30^* flies displayed a wide spectrum of DIOM defects. These mutants often missed DIOMs ranging from none to all of the DIOMs (Figure 3F; n = 5). Therefore, the lack of DIOMs may explain the difficulties of the homozygous *lin-28^dF30^* flies in exiting the pupal case (Movie S1 and S2). A similar spectrum of DIOM defects was also observed in the *lin-28^dF101^* mutants (data not shown).

Our data confirmed the involvement of *let-7* during muscle remodeling, as previously published [23]. The involvement of *let-7* on the muscle phenotype has been already largely described [20], [23]. Therefore, we decided to focus on the fertility decrease exhibited by the homozygous *lin-28^dF30^* flies.

### *lin-28^df30^* Female Mutant Flies Exhibit Reduced Fertility

Deletion of the miRNA *let-7* has been reported to decrease egg laying; however this phenotype has not been studied [23]. Interestingly, Lin28 has recently been suggested to be an important factor in the human ovary germline stem cell (GSC) maintenance [31]. Therefore, we tested if the lack of *lin-28* function also leads to reduced fertility.

**Figure 4:**
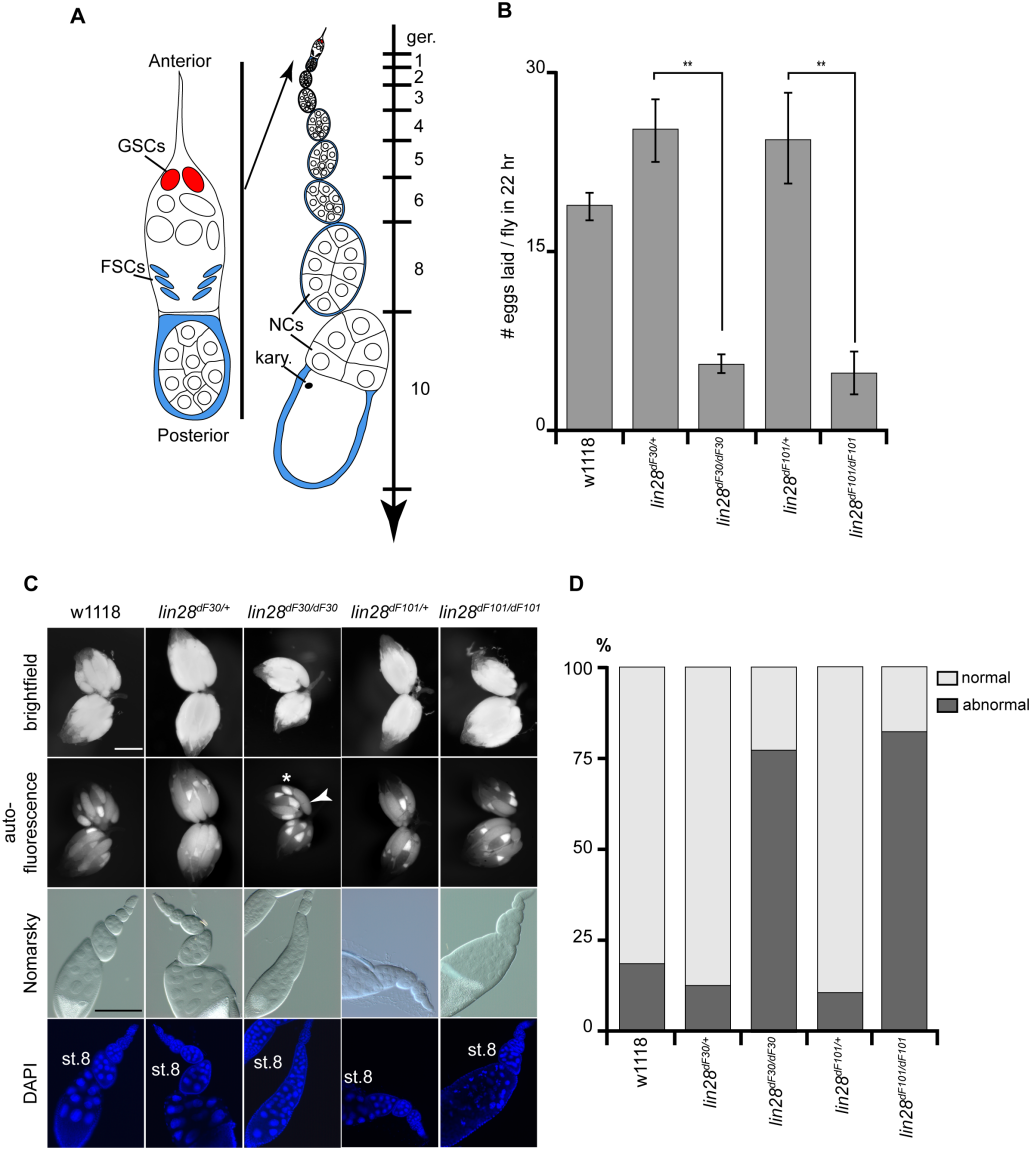
*lin-28* Mutants Exhibit Reduced Fertility. (**A**) Schematic drawing of the germarium with its two stem cell clusters, i.e. germline stem cells (GSCs) and follicular stem cells (FSCs), and of an ovariole composed of a string of egg chambers of increasing maturation stages. The last egg chamber is composed of a large oocyte with its karyosome (kary.) at the posterior pole, and the 15 nurse cells at the anterior pole. (**B**) While *w^1118^* female flies laid 18.8 eggs per day, *lin-28^dF30/+^*25.1 eggs per day and *lin-28^dF101/+^*24.8 eggs per day, *lin-28^dF3^* ^0^ and *lin-28^dF101^* mutant flies laid respectively 5.5 and 5.2 eggs per day (n = 10; p<0.05). (**C**) Comparison of whole ovaries revealed increased auto-fluorescence in homozygous ovaries. The auto-fluorescence is localized at the oocyte portion of the eggs (asterisk), therefore indicating an increased number of eggs between stages 7 to 10, compared to controls. In addition, the visualization of nuclei in single ovarioles revealed an accumulation of supernumerary nurse cells in *lin-28^dF30^* and *lin-28^dF101^* stage 8 egg chambers. (**D**) 75.4% and 78.1% of homozygous *lin-28^dF30^* and *lin-28^dF101^* egg chambers displayed abnormal egg chambers, compared to 16.9% of the *w^1118^* and 13.8% and 12.7% of the heterozygous *lin-28^dF3^* ^0/+ and^ *lin-28^dF101/+^* ovarioles (n = 65). Scale bars represent 100 µm.

Each ovary is composed of several strings of egg chambers, called ovarioles. Each ovariole represents the succession of the different stages of oogenesis (Figure 4A). There are 14 stages in oogenesis and the progression in development occurs from anterior to posterior along the ovariole [32]. Anteriorly the germarium houses the germline and follicular stem cells (GSCs and FSCs). The FSCs differentiate and form the egg follicle, while the GSCs undergo asymmetric cell divisions, where one of the daughter cells becomes the cystoblast and undergoes four mitotic divisions to produce a cyst of 16 cells. This 16-cell cyst is connected by ring canals. Of these cells, 15 will differentiate into nurse cells and one will become the oocyte, which will acquire the posterior-most position in the egg chamber. The oocyte will give rise to the future egg, while the 15 nurse cells will die through apoptosis during stages 12 and 13 [33]. The oocyte nucleus, known as karyosome, is highly compact and much smaller in size compared to the highly endopolyploid nurse cell nuclei.

**Figure 5:**
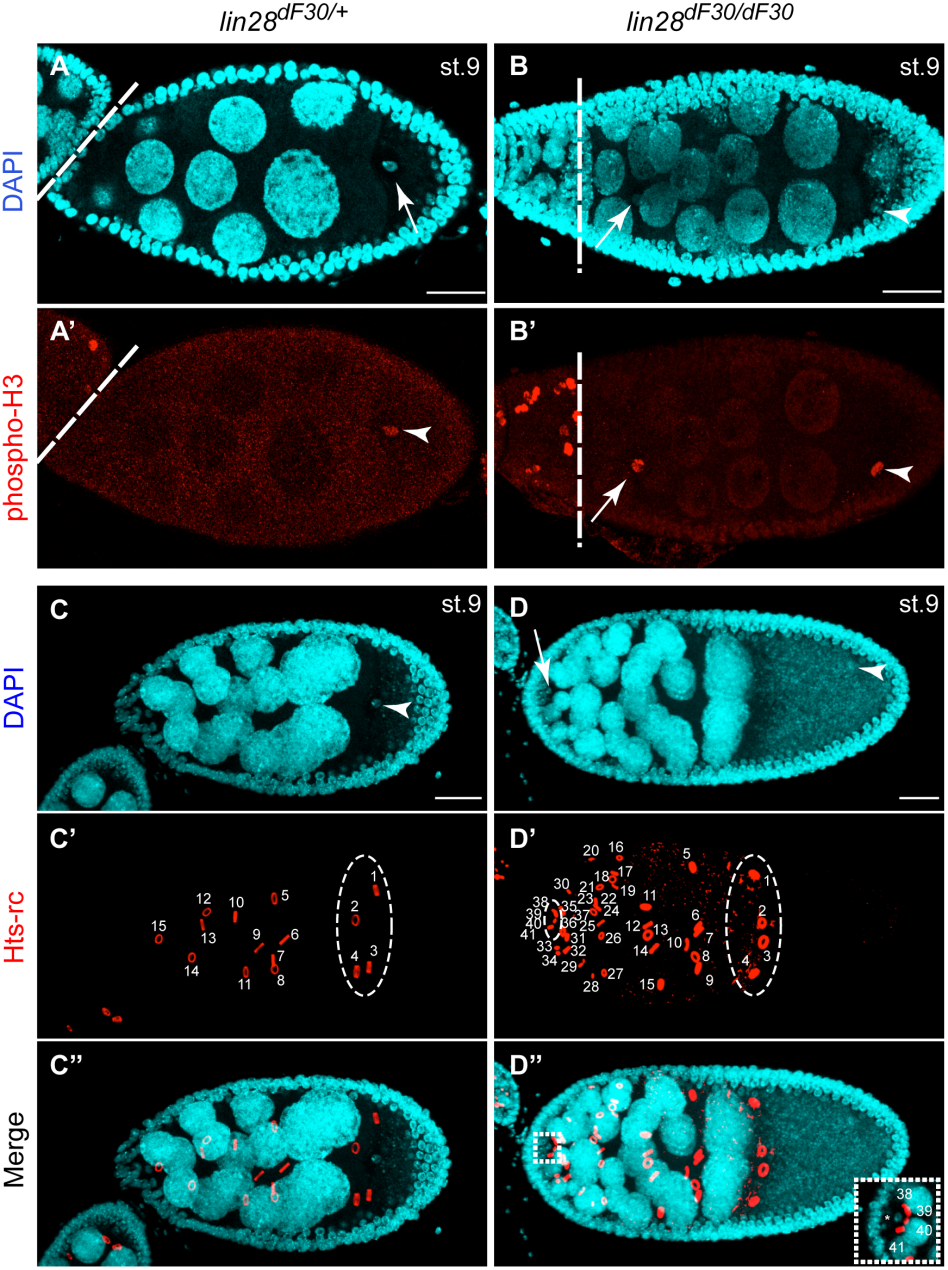
Abnormal Late Egg Chambers Contain Extra Nurse Cells and Extra Oocyte. Homozygous *lin-28^dF30^* displayed an abnormally high number of nurse cells at stage 9, ranging from 22 to 47, compared to the heterozygous *lin-28^dF30/+^* (15 nurse cells). (**A–B’**) In all *lin-28^dF30^* egg chambers that display supernumerary nurse cells, we identified a second karyosome (phospho-H3 positive nucleus, arrow) at the anterior side of the egg chamber (100%; n = 21). (**C–C”**) In *lin-28^dF30/+^* stage 9 egg chambers each of the 15 nurse cells had an associated ring canal and the oocyte was connected to the rest of the egg chamber by four ring canals (circle). (**D–D”**) In *lin-28^dF30^* mutant egg chambers containing supernumerary nurse cells, each nurse cells had a ring canal. In addition, both oocytes (arrows) are connected to the nurse cells by 4 ring canals (circles in **D’**; the insert in **D”** displays a higher magnification of the anterior extra oocyte). Scale bars represent 50 µm.

**Figure 6:**
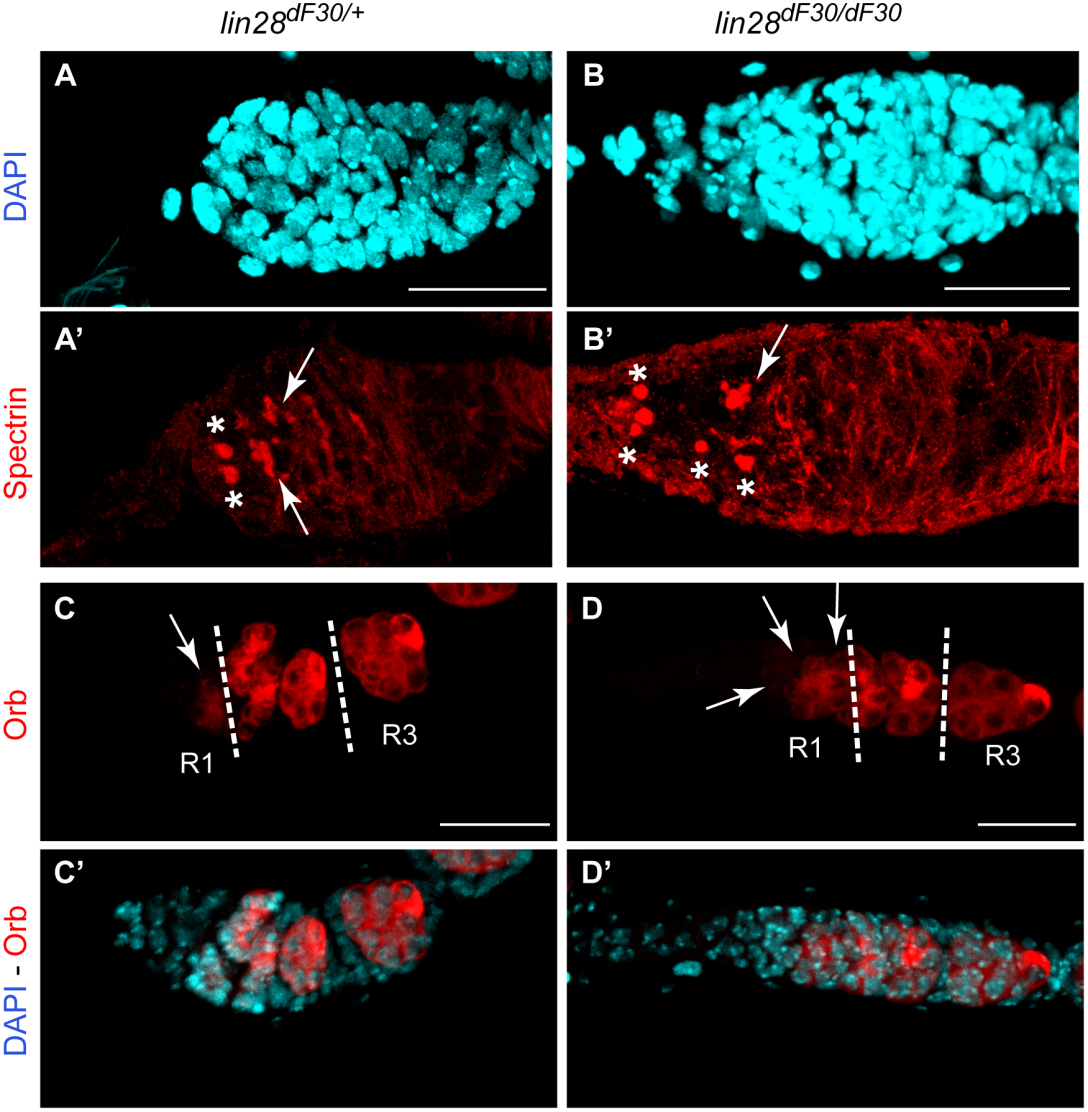
*lin-28^dF30^* Mutant Germaria Suggest Abnormal GSC Differentiation and Early Egg Chamber Fusion. (**A–B’**) Rounded Spectrin marks the GSCs (asterisk), while it stains branched fusomes as the cells differentiate towards the posterior end (arrow) in *lin-28^dF30/+^* germaria (n = 27). In *lin-28^dF30^* germaria, misdifferentiated GSCs retained a round fusome (asterisks) (n = 23). (**C–D’**) Orb expression in the germaria started in R1 (arrow) and was reinforced at stage 2 and 3, when the cyst was encapsulated by follicle cells to form the egg chamber. In *lin-28^dF30^* germaria, more cystoblasts were expressing Orb in R1 (arrow) (76.9%; n = 39). Scale bars represent 25µm.

Interestingly, *lin-28^dF30^* and *lin-28^dF101^* mutant females exhibited reduced egg laying, a phenotype similar to the one described for the *let-7* mutants [23]. Analysis of the *lin-28* phenotype showed that 10 day-old heterozygous *lin-28^dF30/+^* and *lin-28^dF101/+^* females (n = 10) laid about 25 eggs per 22 hours, while homozygous mutant females laid (n = 10) only around 5 eggs during the same time period (Figure 4B).

To understand this drastic difference in egg laying rate, we analyzed the impact of *lin-28* deletion on ovarian morphology. Although the general morphology of the homozygous mutant fly ovaries did not seem affected (Figure 4C), we observed an increase in auto-fluorescence. We found that auto-fluorescence is localized at the oocyte portion of the ovaries. Therefore, we used it as an indirect method to quantify fully formed eggs (dim autofluorescence; Figure 4C - arrowhead) and vitellogenic oocytes (bright auto-fluorescence; Figure 4C - asterisk). The homozygous mutant fly ovaries seemed to have more developing oocytes than formed eggs, compared to heterozygous fly ovaries.

Next, we focused on later stages of oogenesis and analyzed the egg chambers from stage 7 to 10. Whereas ovarioles from wild type and heterozygous flies displayed a normal amount of nurse cells, the homozygous ovarioles had egg chambers with more than 15 nurse cells (and corresponding nuclei) at around stage 8 of oogenesis (Figure 4C). To evaluate the penetrance of this phenotype, we analyzed the ovaries by counting the ovarioles containing egg chambers with larger than normal number of nurse cells or degenerating egg chambers (characterized by the presence of pyknotic nuclei). While 16.9%, 13.8% and 12.7% of the ovarioles contained at least one impaired late egg chamber in the wild type (*w^1118^*) and heterozygous *lin-28^dF30/+^* and *lin-28^dF101/+^* ovaries respectively, 75.4% and 78.1% of homozygous *lin-28^dF30^* and *lin-28^dF101^* ovarioles displayed abnormal egg chambers (Figure 4D; n = 65 for each genotype). Interestingly, we did not observe any abnormal egg chamber after stage 10. Altogether, our observations suggest that the presence of extra nurse cells and arrest of maturation prior to stage 10 in homozygous *lin-28* mutant ovaries can explain, at least partially, the loss of fertility.

### Loss of *lin-28* Affects the Number of Nurse Cells per Egg Chamber

To comprehend the abnormal oogenesis in homozygous *lin-28^dF30^* ovaries, we further analyzed the formation and the development of abnormal egg chambers. Because the late abnormal egg chambers exhibited a large amount of nurse cells, we analyzed the cell proliferation with phospho-Histone 3 (phospho-H3) staining (Figure 5 A–B’). While we did not find any noticeable change in the nurse cell proliferation, the phospho-H3 staining gave us new information about the homozygous *lin-28^dF30^* late oogenesis stages. Due to the staining of condensed chromosomes, phospho-H3 can be used as a marker for karyosome identification (Figure 5A and 5A’ - arrowheads) [34]. Interestingly, in all homozygous *lin-28^dF30^* stage 8 or 9 egg chambers with more than 15 nurse cells we discovered an ectopic karyosome located in an abnormal position on the anterior of the egg chamber (Figure 5B and 5B’ - arrows).

Because each nurse cell is associated with ring canals in the egg chamber, we confirmed the identity of the cells in the egg chamber by the presence of ring canals in the late stage homozygous *lin-28^dF30^* egg chambers (Figure 5C–5D”; n = 21). As expected, we always observed as many ring canals as nuclei in the egg chamber. Moreover, the ectopic oocyte, containing the extra karyosome (Figure 5D - arrows) always had 4 ring canals, similarly to the normal oocyte (Figure 5C’ and 5D’ - circles; 5D” - insert).

Our observations hinted at possible early defects during oogenesis, such as cell proliferation regulation and/or egg chamber fusion. To understand the impact of lin-28 mutation on the ovarian phenotype, we investigated further the oogenesis process.

### *lin-28^df30^* Mutant Flies Display Abnormal GSC Differentiation in the Early Stages of Oogenesis

We hypothesized that the observed phenotype resulted from an early defect during oogenesis, such as GSC differentiation defects and/or egg chamber fusion. Therefore, we analyzed the early GSCs differentiation in the homozygous *lin-28^dF30^* germaria. As it has already been reported, the Spectrin pattern in the germarium reflects the differentiation process of the GSCs [35]. In wild type germaria, the two GSC are characterized by round Spectrin accumulation, while as cells differentiate towards the posterior, Spectrin is involved in the formation of the fusome and it acquires a branched morphology. The fusome is a germline specific structure with branching arms that extend through each intercellular bridge in the cyst [36]. A normal GSC differentiation process was visible in the heterozygous *lin-28^dF30/+^* germaria (Figure 6A and 6A’). The rounded fusome in the GSCs (Figure 6A’, asterisks) changed to a branched structure (Figure 6A’ - arrow). However, 32% (n = 23) of the homozygous *lin-28^dF30^* germaria displayed an increase of undifferentiated GSCs visible through their rounded Spectrin pattern. Apart from the two GSCs next to the cap cells, extra cells contained a rounded fusome posteriorly to the GSCs (Figure 6B and 6B’ - asterisks; Movie S3). This increase of undifferentiated cells delayed the appearance of a branched fusome (Figure 6B’ - arrow) and delayed the first stage of oogenesis.

The rounded fusome confirmed early GCS differentiation defects. To further validate the possibility of an early egg chamber fusion, we analyzed early expression of *Orb* (Figure 6C–6D’). The expression pattern of *Orb* during oogenesis has been already extensively described, and its early expression in the cyst allows tracking how many cysts are engulfed in the same egg chamber [37]–[39]. In heterozygote germaria only one or two cells expressed *Orb* in region 1 (R1) (Figure 6C - arrow) but in homozygous *lin-28^dF30^* germaria, *Orb* was expressed by several cystoblasts in R1 (Figure 6D - arrow; 76.9%, n = 39). This could already reflect the phenotype seen more clearly at later stages (Figure 4C and Figure 5) *i.e.* packing of two cysts in the same egg chamber.

Altogether our results point out GSC differentiation defects and probable early egg chamber fusion that lead to late abnormal egg chambers and reduced fertility.

### Fused Egg Chamber Phenotype is *let-7* Dependent

To further characterize the dependency of the ovarian phenotype to the *lin-28* mutation, we analyzed the penetrance of the phenotype upon various alterations of Lin-28 or *let-7*.

**Figure 7:**
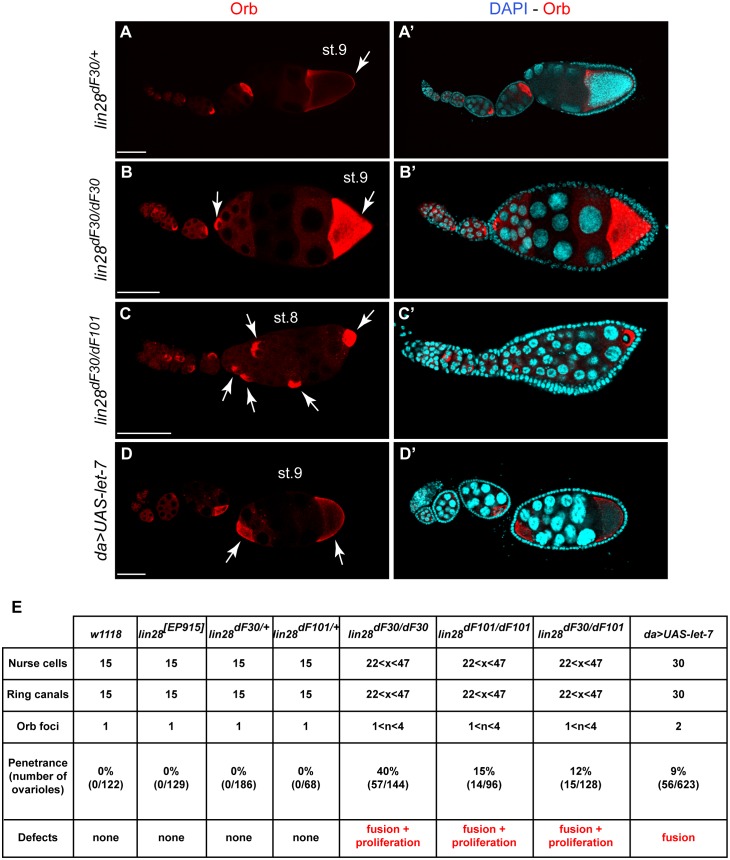
Egg Chambers Containing Extra Nurse Cells Display Several Orb Foci. (**A–A’**) During normal oogenesis, Orb is located at the posterior pole of the maturing egg chamber (arrowhead). (**B–B’**) In *lin-28^dF30^* stage 9 abnormal egg chambers, a secondary Orb locus is found at the anterior pole (arrow). (**C–C’**) Multiple Orb foci were seen in the trans heterozygous *lin-28^dF30/dF101^*. Such Orb expression pattern was also present in *lin-28^dF101/dF101^*, but not shown. (**D–D’**) The egg chamber fusion phenotype has been recapitulated by the ectopic expression of *let-7* in follicle cells. (**E**) The penetrance of the egg chamber fusion and abnormal proliferation has been summarized in a table. Scale bars represent 50 µm.

Heterozygous *lin-28^dF30/+^* late egg chambers exhibited one developing oocyte characterized by Orb expression (Figure 7A and 7A’; 100%, n = 186)In homozygous *lin-28^dF30^* abnormal late egg chambers, we observed two developing oocytes (Figure 7B and 7B’; 40%, n = 144). Few late egg chambers displayed a third or more Orb foci (data not shown). These abnormal egg chambers displayed from 22 to 47 nurse cells, each one having one ring canal. This observation hinted at a defect of mitosis. The same observation was made in the homozygous *lin-28^dF101^* abnormal late egg chambers (data not shown). In order to rule out a secondary mutation site during imprecise P-element excision, we analyzed the *lin-28^dF30/dF101^* trans-heterozygous abnormal late egg chambers, and observed the same phenotype (Figure 7C and 7C’; 12%, n = 128). Notably, we never observed more than one oocyte in any of the control tested, namely wild type (*w^1118^*), homozygous *lin^[EP915]^* (P element line used for original excisions) and heterozygous (*lin-28^dF30/+^* and *lin-28^dF101/+^*) ovaries (data not shown).

Lin-28 has been shown to exert its action through physical interaction and consequent inhibition of the *let-7* miRNA. Therefore, we investigated if the supernumerary nurse cell nuclei phenotype that results after *lin-28* deletion is related to *let-7* upregulation, we expressed the *UAS-let-7* construct under the ubiquitous *da-GAL4* promoter. However, the UAS-GAL4 system has a limited action in the germline cells during oogenesis [40]. To confirm this, we used a nuclear GFP reporter and found that the *da-GAL4>UAS-nGFP* expression was below the detection limit in the germline (data not shown). Therefore, in *da-GAL4>UAS-let-7* ovaries, *let-7* is ectopically expressed in follicle cells. Interestingly, in these flies the egg chamber fusion phenotype was recapitulated (Figure 7D and 7D’; 9%, n = 623), while we could not recapitulate the mitosis defect (always 30 nurse cells). Based on these results, we propose that *let-7* overexpression in follicle cells results in an early egg chamber fusion. On the other hand, the mitosis phenotype is either germ cell-related, or alternatively is follicle cell-related, but not *let-7* related. We summarized our observations in a table (Figure 7E).

**Figure 8:**
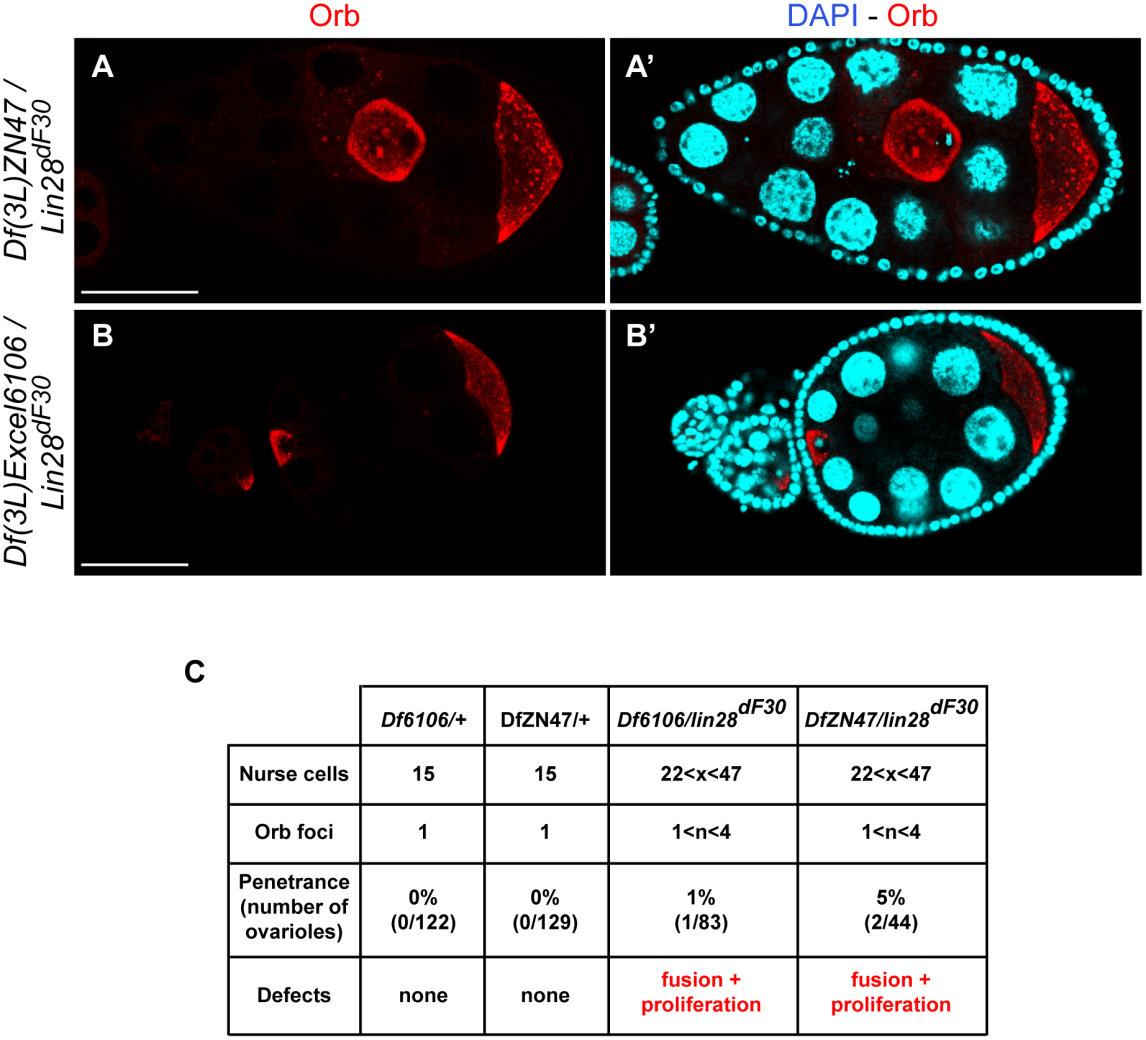
*lin28* Mutation is Responsible for Abnormal Egg Chamber Phenotype. The recapitulation of the abnormal egg chamber phenotype was obtained with a low penetrance by complementing our *Lin28^dF30^* allele with available deficiencies. (**A–B’**) *Df(3L)ZN47/Lin28^dF30^* (5%; n = 44) and *Df(3L)Exel6106/Lin28^dF30^* (1%; n = 83) displayed supernumerary nurse cells and extra Orb foci in late egg chambers. (**C**) The penetrance of the phenotype has been summarized in a table. Scale bars: 50 µm.

To insure the Lin-28-dependency of the phenotype we obtained, we complemented the *lin-28^dF30^* allele with two deficiency lines available, Df(3L)ZN47 and Df(3L)Excel6106. While we recapitulated the homozygous *Lin28^dF30^* ovarian phenotype (Figure 8), the phenotype penetrance was drastically lower: 1% (n = 83) in *Df(3L)Exel6106/Lin28^dF30^* ovaries and 5% (n = 44) in *Df(3L)ZN47/Lin28^dF30^* ovaries. Interestingly the deficiency line alleles bear deletions of several genes, including *lin-28* and *Blimp-1*. Blimp-1 is a highly conserved transcription factor involved in mammalian germline cell progeny segregation [41]. Moreover, *Blimp-1* is a target of *let-7* and it is involved in the *Lin28/let-7* regulatory network [42]. We hypothesized that the discrepancies observed probably resulted from the interaction between Blimp-1 and the Lin28/let-7 network.

Our data suggested a compound phenotype with mitotic defect and egg chamber fusion, as reported earlier in the *maelstrom* mutant line [43]. Moreover, this compound phenotype was always recapitulated when decreasing Lin-28 regulatory activity or increasing *let-7* expression. Therefore, this phenotype seems to be Lin-28-dependent.

### Abnormal EcR Expression Pattern in Homozygous *lin-28^df30^* Abnormal Late Egg Chambers

**Figure 9:**
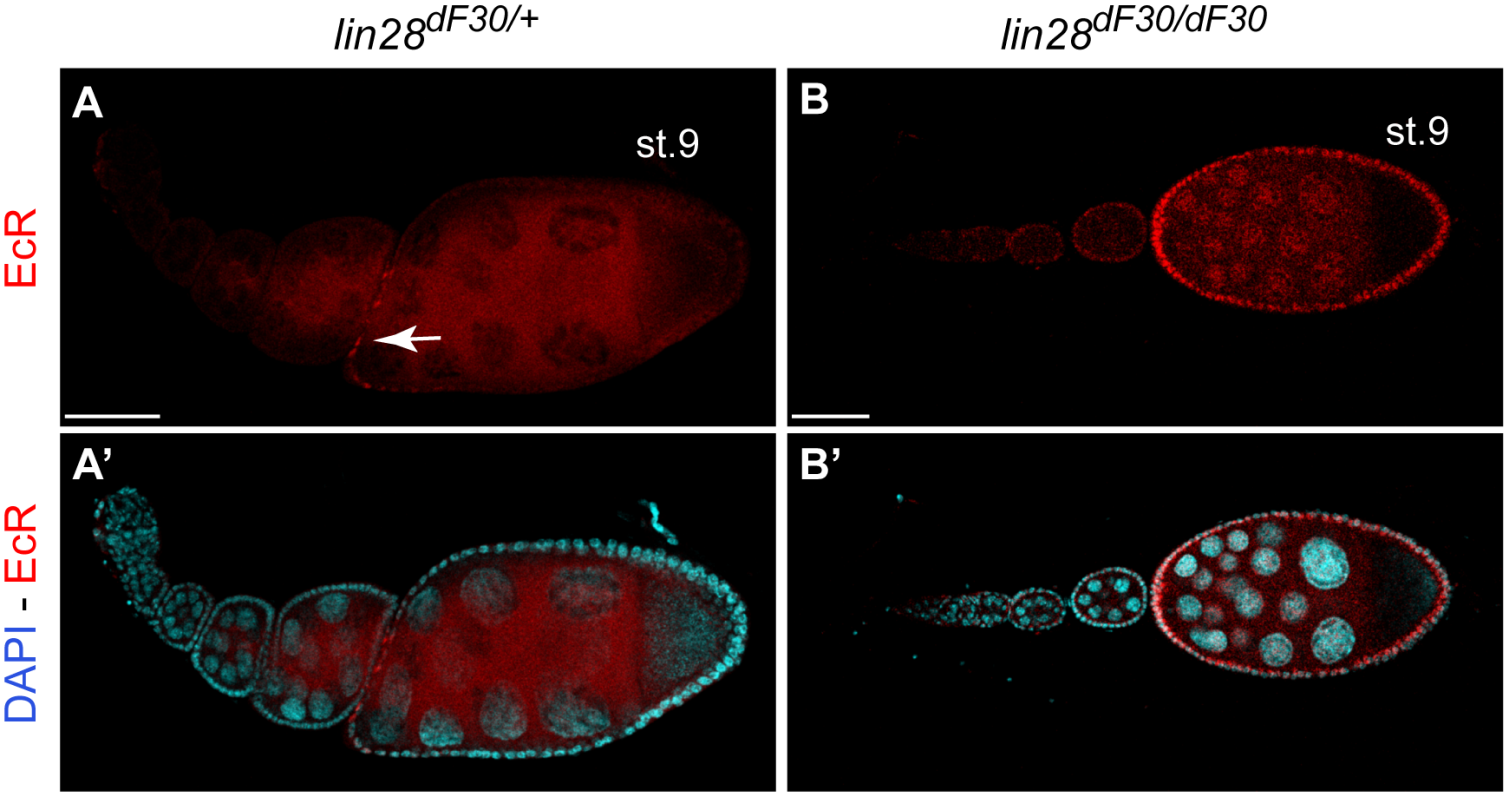
*lin-28^dF30^* Abnormal Egg Chambers Present Abnormal EcR Expression Pattern. (**A–B’**) Ecdysone Receptor (EcR) expression was detected only in anterior follicle cells of late egg chamber (arrow), while in all *lin-28^dF30^* abnormal egg chambers (n = 42), EcR was expressed by all follicle cells. Scale bars represent 50 µm.

In order to further characterize the follicle cells of fused late egg chambers, we used Ecdysone Receptor (EcR) staining (Figure 9; n = 42). *EcR* is expressed at stage 9 exclusively in the anterior follicle cells [24]. While this pattern was found in the heterozygous *lin-28^dF30/+^* stage 9 egg chambers (Figure 9A - arrow), we found a homogenous follicular *EcR* expression in all abnormal homozygous *lin-28^dF30^* mutant stage 9 egg chambers (Figure 9B). Therefore, we conclude that despite the fusion, the follicle cells of abnormal *lin-28^dF30^* egg chambers are homogenously expressing EcR. Consequently, the EcR ectopic expression in late egg chambers, may lead to abnormal egg maturation due to misregulated Ecdysone signalling.

### Lin28/let-7 Regulates Abrupt/Fas2 Network during Oogenesis

Because of the increase of *let-7* maturation in homozygous *lin-28^dF30^* ovaries (Figure 1D), we suspected a drastic misregulation of *let-7* targets during oogenesis. Therefore, we searched for the predicted targets for *dme-let-7* from the TargetScanFly (http://www.targetscan.org/fly_12/) and miRBase (http://www.mirbase.org) databases. Two genes among the predicted targets are involved in oogenesis: *Stet* and *Abrupt*. While Stet has not been studied in this context, *Abrupt* has been shown to be a *let-7* target *in vivo* [20]. Moreover Abrupt is involved in border cell (BC) migration in the late stage of oogenesis [24] and it has been shown to repress expression of the cell adhesion molecule *Fas2* in the *Drosophila* developing brain [26].

**Figure 10:**
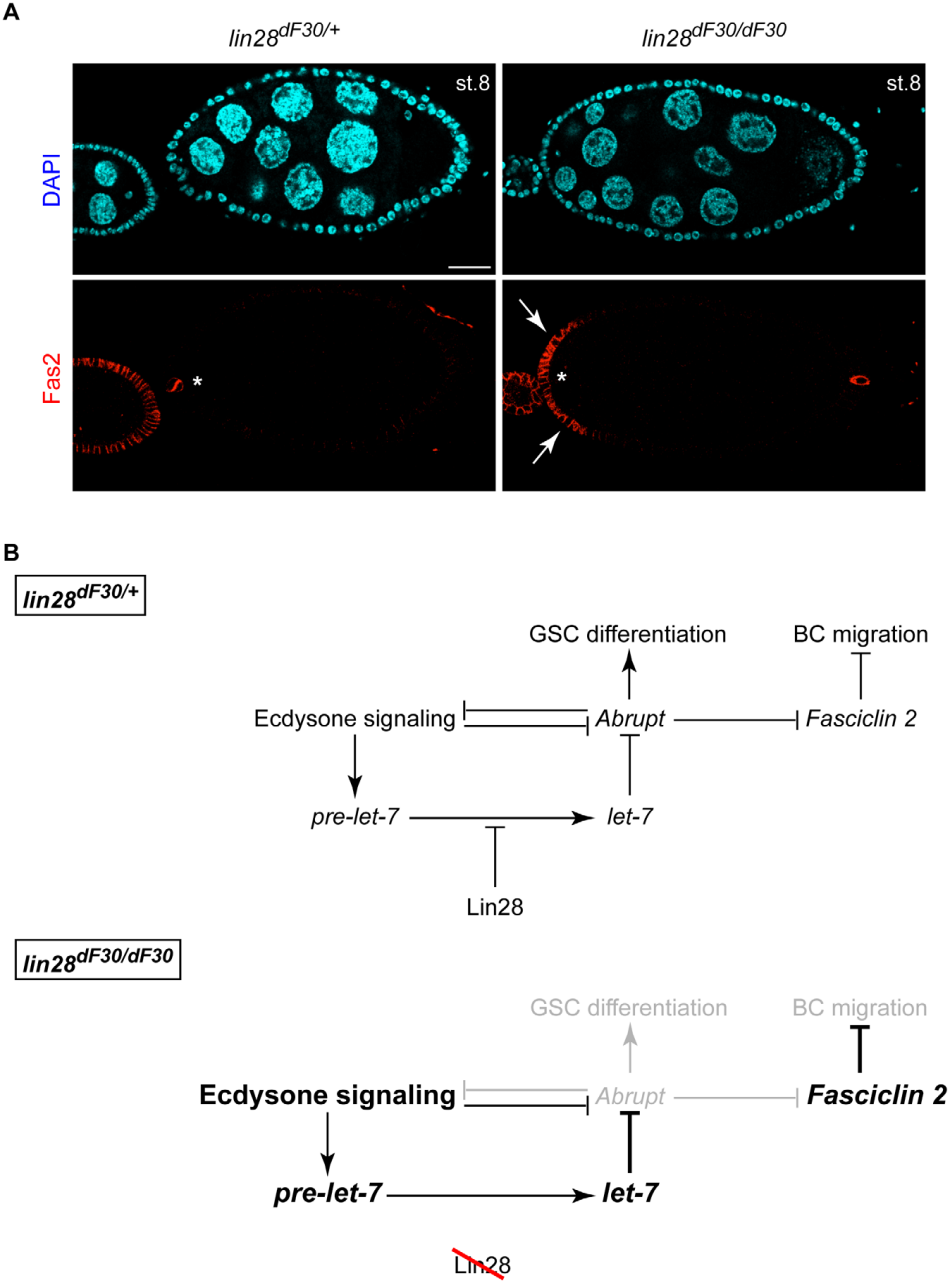
*lin-28^dF30^* Abnormal Egg Chambers Exhibit Impaired Fas2 Expression Pattern, which is Necessary during Oogenesis. (**A**) While Fas2 expression was confined to the polar cells in *lin-28^dF30/+^* stage 8 egg chamber (asterisk), all (n = 38) the *lin-28^dF30^* stage 8 abnormal egg chambers displayed an extended anterior expression domain (arrows). (**B**) Abrupt has a pivotal role during oogenesis, taking part in GSC differentiation and regulating BC migration. The proposed network could explain the various defects observed in the *lin-28^dF30^* ovaries. Scale bar represent 25 µm.

Therefore, we investigated *Fas2* expression in abnormal egg chambers (Figure 10; n = 38). *Fas2* is expressed in all follicle cells through stage 7. At stage 8 and specifically when BCs differentiate preceding cell cluster delamination, *Fas2* expression is lost in all anterior follicle cells, including the BCs, and it is only expressed in the polar cells. This differential pattern of expression leads to a polarity switch in the polar cells, which triggers BC delamination from the follicle [44]. Strikingly, while heterozygous *lin-28^dF30/+^* ovarioles displayed a *Fas2* expression similar to wild type (Figure 10A), all the homozygous *lin-28^dF30^* stage 8 and 9 egg chambers containing supernumerary nurse cells exhibited an ectopic *Fas2* expression at the anterior part of the egg chambers (arrows), compromising the border cell migration. Indeed, we never observed the migration of border cells in abnormal late egg chambers (data not shown).

This result suggested the interaction of the *Abrupt/Fas2* network with *lin-28/let-7* during oogenesis (Figure 10B).

## Discussion

Because of their role during stem cell differentiation, members of the *let-7* miRNA family have been extensively studied. However, the role of *lin-28* is still poorly documented.

Deletion of *let-7* in *Drosophila* impairs the musculature remodeling during the larva to adult metamorphosis. For instance the DIOMs, muscles which are required for eclosion and which are lost within 12 hours after eclosion, they are maintained during adulthood upon *let-7* deletion [23]. By generating the first *lin-28* deletion in flies, we successfully confirmed the involvement of Lin-28/let-7 regulatory network in DIOM remodeling. In this study, we showed that deletion of *lin-28*, led to over maturation of *let-7*, which negatively affected, and sometimes prevented DIOM formation. This drastic phenotype led to a suboptimal muscular phenotype. However, due to a variable penetrance of the *lin-28* deletion phenotype, a proportion of the flies could eclose and live as fertile animals.

In addition, we discovered a link between Lin-28 function and oogenesis. Our data indicates a role of *let-7* during GSC differentiation and egg chamber formation. Because of the importance of these processes, *let-7* maturation has to be strictly regulated by Lin-28 activity. We suggest that a potential network involving Lin-28/*let-7*/Ecdysone signaling/Abrupt/Fas2 is needed during GSC differentiation and BC migration (Figure 10B). The role of Abrupt in downregulating the steroid hormone Ecdysone has previously been demonstrated [45]. Indeed, the loss of Taiman, a target of the transcription factor Abrupt and co-activator of Ecdysone receptor, leads to an increase of undifferentiated GSCs in the germarium due to disruption of Ecdysone signaling [45], [46]. Therefore, by regulating the expression pattern of *Abrupt*, Lin28/*let-7* may adjust the domain of Ecdysone activity, providing a control over the GSCs differentiation and egg chamber maturation during the oogenesis. Indeed, it has been shown that the Ecdysone titre rises during oogenesis at stage 9 [47]. While the precise Ecdysone expression pattern is not known, we suggest that the uniform *EcR* expression pattern in follicle cells in *lin-28* mutants may break the Ecdysone signaling asymmetry needed during proper oogenesis.

Furthermore, a previous study demonstrated the activation of *let-7* expression via Ecdysone activity [22]. In this study, we showed that *lin-28* deletion, resulted in the alleviation of Lin28’s inhibitory role on *let-7* maturation. This led to loss of Abrupt, which in turn inhibited Ecdysone activity and maintained *Fas2* expression, resulting in BC migration impairment. To test whether the increase of Ecdysone signaling amplifies *let-7* expression through a positive feedback loop [22], we generated a system in which there is no control of either *let-7* expression nor of Ecdysone activity. This situation leads to an early cyst fusion, a loss of proper GSC differentiation and a mitotic defect, as we observed in the homozygous *lin-28^dF30^* ovaries. The accumulation of these defects may be enough to trigger apoptosis at mid-oogenesis, a well-known checkpoint previously described [48].

Interestingly, the variable penetrance of the phenotype allows proper oogenesis and appearance of subfertile adult flies. This suggests a robust molecular network where feedback loops can rescue the system if one component disturbs the balance.

## Conclusions

By combining our results with previously published studies, we suggest an conserved link between hormonal signaling and germline stem cell differentiation, involving the *let-7* miRNA family. This suggestion is reinforced in the last couple of years by the discovery of dormant ovarian follicles and mitotically active germ cells in adult mammalian ovaries, which are responsive to gonadotropin hormone [49]–[51]. Moreover, it has been demonstrated that Lin-28 is involved in germline stem cell regulation in human ovary [31] and in the ovarian surface epithelium of severe ovarian infertility patients [52].

## Supporting Information

Movie S1 ***lin-28^dF30^*** **Pharate Adults Fail to Eclose from their Pupal Case.** Although the animal is moving its legs, head and proboscis, it was unable to exit its pupal case (n = 10).
(MOV)

Movie S2 ***lin-28^dF30^*** **Pharate Adults Liberated from the Pupal Case Fail to Walk.** *lin-28^dF30^* homozygous mutants that had problems eclosing were removed from the pupal case with the help of forceps. *lin-28^dF30^* homozygous mutants exhibited abnormal behaviors such as moving only 3 of its 6 legs, while the other 3 were dragged along. In addition, the movements of the fly were slow and impaired. Note, that in Movie S1 and Movie S2, the same animal has been filmed.
(MOV)

Movie S3 ***lin-28^dF30^*** **Germarium Displayed Ectopic Rounded Spectrin Expression Pattern.** The two GSCs next to the cap cells (right side of the movie) contained a rounded fusome. Moreover, three extra rounded fusomes can be seen posteriorly to the GSCs (rounded fusomes are indicated by arrows).
(AVI)

## References

[1] Ambros V, Horvitz HR (1984) Heterochronic mutants of the nematode Caenorhabditis elegans. Science 226: 409–416. 6494891 10.1126/science.6494891

[2] Fire A, Xu S, Montgomery MK, Kostas SA, Driver SE, et al (1998) Potent and specific genetic interference by double-stranded RNA in Caenorhabditis elegans. Nature 391: 806–811. 9486653 10.1038/35888

[3] Reinhart BJ, Slack FJ, Basson M, Pasquinelli AE, Bettinger JC, et al (2000) The 21-nucleotide let-7 RNA regulates developmental timing in Caenorhabditis elegans. Nature 403: 901–906. 10706289 10.1038/35002607

[4] Yang DH, Moss EG (2003) Temporally regulated expression of Lin-28 in diverse tissues of the developing mouse. Gene Expr Patterns 3: 719–726. 14643679 10.1016/s1567-133x(03)00140-6

[5] Viswanathan SR, Daley GQ, Gregory RI (2008) Selective blockade of microRNA processing by Lin28. Science 320: 97–100. 18292307 10.1126/science.1154040 PMC3368499

[6] Piskounova E, Polytarchou C, Thornton JE, Lapierre RJ, Pothoulakis C, et al (2011) Lin28A and Lin28B Inhibit let-7 MicroRNA Biogenesis by Distinct Mechanisms. Cell 147: 1066–1079. 22118463 10.1016/j.cell.2011.10.039 PMC3227872

[7] Newman MA, Thomson JM, Hammond SM (2008) Lin-28 interaction with the Let-7 precursor loop mediates regulated microRNA processing. RNA 14: 1539–1549. 18566191 10.1261/rna.1155108 PMC2491462

[8] Loughlin FE, Gebert LF, Towbin H, Brunschweiger A, Hall J, et al (2012) Structural basis of pre-let-7 miRNA recognition by the zinc knuckles of pluripotency factor Lin28. Nat Struct Mol Biol 19: 84–89. 10.1038/nsmb.2202 22157959

[9] Zhong X, Li N, Liang S, Huang Q, Coukos G, et al (2010) Identification of microRNAs regulating reprogramming factor LIN28 in embryonic stem cells and cancer cells. J Biol Chem 285: 41961–41971. 20947512 10.1074/jbc.M110.169607 PMC3009922

[10] Yu J, Vodyanik MA, Smuga-Otto K, Antosiewicz-Bourget J, Frane JL, et al (2007) Induced pluripotent stem cell lines derived from human somatic cells. Science 318: 1917–1920. 18029452 10.1126/science.1151526

[11] Richards M, Tan SP, Tan JH, Chan WK, Bongso A (2004) The transcriptome profile of human embryonic stem cells as defined by SAGE. Stem Cells 22: 51–64. 14688391 10.1634/stemcells.22-1-51

[12] Jin J, Jing W, Lei XX, Feng C, Peng S, et al (2011) Evidence that Lin28 stimulates translation by recruiting RNA helicase A to polysomes. Nucleic Acids Res 39: 3724–3734. 21247876 10.1093/nar/gkq1350 PMC3089476

[13] Polesskaya A, Cuvellier S, Naguibneva I, Duquet A, Moss EG, et al (2007) Lin-28 binds IGF-2 mRNA and participates in skeletal myogenesis by increasing translation efficiency. Genes Dev 21: 1125–1138. 17473174 10.1101/gad.415007 PMC1855237

[14] Nam Y, Chen C, Gregory RI, Chou JJ, Sliz P (2011) Molecular Basis for Interaction of let-7 MicroRNAs with Lin28. Cell 147: 1080–91. 22078496 10.1016/j.cell.2011.10.020 PMC3277843

[15] Pasquinelli AE, Reinhart BJ, Slack F, Martindale MQ, Kuroda MI, et al (2000) Conservation of the sequence and temporal expression of let-7 heterochronic regulatory RNA. Nature 408: 86–89. 11081512 10.1038/35040556

[16] Wulczyn FG, Smirnova L, Rybak A, Brandt C, Kwidzinski E, et al (2007) Post-transcriptional regulation of the let-7 microRNA during neural cell specification. FASEB J 21: 415–426. 17167072 10.1096/fj.06-6130com

[17] Rybak A, Fuchs H, Hadian K, Smirnova L, Wulczyn EA, et al (2009) The let-7 target gene mouse lin-41 is a stem cell specific E3 ubiquitin ligase for the miRNA pathway protein Ago2. Nat Cell Biol 11: 1411–1420. 19898466 10.1038/ncb1987

[18] Li X, Zhang J, Gao L, McClellan S, Finan MA, et al (2011) MiR-181 mediates cell differentiation by interrupting the Lin28 and let-7 feedback circuit. Cell Death Differ 19: 378–86. 21979467 10.1038/cdd.2011.127 PMC3278736

[19] Roush S, Slack FJ (2008) The let-7 family of microRNAs. Trends Cell Biol 18: 505–516. 18774294 10.1016/j.tcb.2008.07.007

[20] Caygill EE, Johnston LA (2008) Temporal regulation of metamorphic processes in Drosophila by the let-7 and miR-125 heterochronic microRNAs. Curr Biol 18: 943–950. 18571409 10.1016/j.cub.2008.06.020 PMC2736146

[21] Sempere LF, Dubrovsky EB, Dubrovskaya VA, Berger EM, Ambros V (2002) The expression of the let-7 small regulatory RNA is controlled by ecdysone during metamorphosis in Drosophila melanogaster. Dev Biol 244: 170–179. 11900466 10.1006/dbio.2002.0594

[22] Chawla G, Sokol NS (2012) Hormonal activation of let-7-C microRNAs via EcR is required for adult Drosophila melanogaster morphology and function. Development 139: 1788–1797. 22510985 10.1242/dev.077743 PMC3328179

[23] Sokol NS, Xu P, Jan YN, Ambros V (2008) Drosophila let-7 microRNA is required for remodeling of the neuromusculature during metamorphosis. Genes Dev 22: 1591–1596. 18559475 10.1101/gad.1671708 PMC2428057

[24] Jang AC, Chang YC, Bai J, Montell D (2009) Border-cell migration requires integration of spatial and temporal signals by the BTB protein Abrupt. Nat Cell Biol 11: 569–579. 19350016 10.1038/ncb1863 PMC2675665

[25] Ahn HW, Morin RD, Zhao H, Harris RA, Coarfa C, et al (2010) MicroRNA transcriptome in the newborn mouse ovaries determined by massive parallel sequencing. Mol Hum Reprod 16: 463–471. 20215419 10.1093/molehr/gaq017 PMC2882868

[26] Kucherenko MM, Barth J, Fiala A, Shcherbata HR (2012) Steroid-induced microRNA let-7 acts as a spatio-temporal code for neuronal cell fate in the developing Drosophila brain. EMBO J 31: 4511–23. 23160410 10.1038/emboj.2012.298 PMC3545287

[27] Wodarz A, Hinz U, Engelbert M, Knust E (1995) Expression of crumbs confers apical character on plasma membrane domains of ectodermal epithelia of Drosophila. Cell 82: 67–76. 7606787 10.1016/0092-8674(95)90053-5

[28] Michon F, Tummers M, Kyyrönen M, Frilander MJ, Thesleff I (2010) Tooth morphogenesis and ameloblast differentiation are regulated by micro-RNAs. Dev Biol 340: 355–368. 20102707 10.1016/j.ydbio.2010.01.019

[29] Stockinger P, Kvitsiani D, Rotkopf S, Tirián L, Dickson BJ (2005) Neural circuitry that governs Drosophila male courtship behavior. Cell 121: 795–807. 15935765 10.1016/j.cell.2005.04.026

[30] Coulom H, Birman S (2004) Chronic exposure to rotenone models sporadic Parkinson’s disease in Drosophila melanogaster. J Neurosci 24: 10993–10998. 15574749 10.1523/JNEUROSCI.2993-04.2004 PMC6730201

[31] Childs A, Kinnell HL, He J, Anderson RA (2012) LIN28 is selectively expressed by primordial and pre-meiotic germ cells in the human fetal ovary. Stem Cells Dev 21: 2343–9. 22296229 10.1089/scd.2011.0730 PMC3424972

[32] White RA, Perrimon N, Gehring WJ (1984) Differentiation markers in the Drosophila ovary. J Embryol Exp Morphol 84: 275–286. 6442733

[33] Foley K, Cooley L (1998s) Apoptosis in late stage Drosophila nurse cells does not require genes within the H99 deficiency. Development 125: 1075–1082. 9463354 10.1242/dev.125.6.1075

[34] Djagaeva I, Doronkin S, Beckendorf SK (2005) Src64 is involved in fusome development and karyosome formation during Drosophila oogenesis. Dev Biol 284: 143–156. 15979065 10.1016/j.ydbio.2005.05.012

[35] de Cuevas M, Lee JK, Spradling AC (1996) alpha-spectrin is required for germline cell division and differentiation in the Drosophila ovary. Development 122: 3959–3968. 9012516 10.1242/dev.122.12.3959

[36] Li Y, Maines JZ, Tastan OY, McKearin DM, Buszczak M (2012) Mei-P26 regulates the maintenance of ovarian germline stem cells by promoting BMP signaling. Development 139: 1547–56. 22438571 10.1242/dev.077412 PMC3317963

[37] Lantz V, Chang JS, Horabin JI, Bopp D, Schedl P (1994) The Drosophila orb RNA-binding protein is required for the formation of the egg chamber and establishment of polarity. Genes Dev 8: 598–613. 7523244 10.1101/gad.8.5.598

[38] O’Reilly AM, Ballew AC, Miyazawa B, Stocker H, Hafen E, et al (2006) Csk differentially regulates Src64 during distinct morphological events in Drosophila germ cells. Development 133: 2627–2638. 16775001 10.1242/dev.02423

[39] Wong LC, Costa A, McLeod I, Sarkeshik A, Yates J, et al (2011) The functioning of the Drosophila CPEB protein Orb is regulated by phosphorylation and requires casein kinase 2 activity. PLoS One 6: e24355. 21949709 10.1371/journal.pone.0024355 PMC3176278

[40] Rørth P (1998) Gal4 in the Drosophila female germline. Mech Dev 78: 113–118. 9858703 10.1016/s0925-4773(98)00157-9

[41] Hopf C, Viebahn C, Püschel B (2011) BMP signals and the transcriptional repressor BLIMP1 during germline segregation in the mammalian embryo. Dev Genes Evol 221: 209–223. 21881976 10.1007/s00427-011-0373-5 PMC3192270

[42] West JA, Viswanathan SR, Yabuuchi A, Cunniff K, Takeuchi A, et al (2009) A role for Lin28 in primordial germ-cell development and germ-cell malignancy. Nature 460: 909–913. 19578360 10.1038/nature08210 PMC2729657

[43] Sato K, Nishida KM, Shibuya A, Siomi MC, Siomi H (2011) Maelstrom coordinates microtubule organization during Drosophila oogenesis through interaction with components of the MTOC. Genes Dev 25: 2361–2373. 22085963 10.1101/gad.174110.111 PMC3222902

[44] Szafranski P, Goode S (2004) A Fasciclin 2 morphogenetic switch organizes epithelial cell cluster polarity and motility. Development 131: 2023–2036. 15056617 10.1242/dev.01097

[45] König A, Yatsenko AS, Weiss M, Shcherbata HR (2011) Ecdysteroids affect Drosophila ovarian stem cell niche formation and early germline differentiation. EMBO J 30: 1549–1562. 21423150 10.1038/emboj.2011.73 PMC3102283

[46] Jang AC, Chang YC, Bai J, Montell D (2009) Border-cell migration requires integration of spatial and temporal signals by the BTB protein Abrupt. Nat Cell Biol 11: 569–579. 19350016 10.1038/ncb1863 PMC2675665

[47] Schwartz MB, Kelly TJ, Woods CW, Imberski RB (1989) Ecdysteroid fluctuations in adult <i> Drosophila melanogaster caused by elimination of pupal reserves and synthesis by early vitellogenic ovarian follicles. Insect biochemistry 19: 243–249.

[48] Pritchett TL, Tanner EA, McCall K (2009) Cracking open cell death in the Drosophila ovary. Apoptosis 14: 969–979. 19533361 10.1007/s10495-009-0369-z PMC2810646

[49] Li J, Kawamura K, Cheng Y, Liu S, Klein C, et al (2010) Activation of dormant ovarian follicles to generate mature eggs. Proc Natl Acad Sci U S A 107: 10280–10284. 20479243 10.1073/pnas.1001198107 PMC2890455

[50] Bhartiya D, Sriraman K, Gunjal P, Modak H (2012) Gonadotropin treatment augments postnatal oogenesis and primordial follicle assembly in adult mouse ovaries? J Ovarian Res 5: 32. 23134576 10.1186/1757-2215-5-32 PMC3616927

[51] White YA, Woods DC, Takai Y, Ishihara O, Seki H, et al (2012) Oocyte formation by mitotically active germ cells purified from ovaries of reproductive-age women. Nat Med 18: 413–421. 22366948 10.1038/nm.2669 PMC3296965

[52] Virant-Klun I, Skutella T, Stimpfel M, Sinkovec J (2011) Ovarian surface epithelium in patients with severe ovarian infertility: a potential source of cells expressing markers of pluripotent/multipotent stem cells. J Biomed Biotechnol 2011: 381928. 22187524 10.1155/2011/381928 PMC3237017

